# Kerf Taper Defect Minimization Based on Abrasive Waterjet Machining of Low Thickness Thermoplastic Carbon Fiber Composites C/TPU

**DOI:** 10.3390/ma12244192

**Published:** 2019-12-13

**Authors:** Alejandro Sambruno, Fermin Bañon, Jorge Salguero, Bartolome Simonet, Moises Batista

**Affiliations:** 1 Department of Mechanical Engineering and Industrial Design, Faculty of Engineering, University of Cadiz, Av. Universidad de Cadiz 10, E-11519 Puerto Real, Cadiz, Spain; fermin.banon@uca.es (F.B.); jorge.salguero@uca.es (J.S.); moises.batista@uca.es (M.B.); 2 Nanotures SL, C. Inteligencia 19, Tecnoparque Agroalimentario, E-11591 Jerez de la Frontera, Cadiz, Spain; bartolome.simonet@nanotures.es

**Keywords:** AWJM (Abrasive waterjet machining), CFRTP (Carbon fiber-reinforced thermoplastics), kerf taper, RSM (response surface methodology), ANOVA (Analysis of variance), C/TPU (carbon/polyurethane)

## Abstract

Carbon fiber-reinforced thermoplastics (CFRTPs) are materials of great interest in industry. Like thermosets composite materials, they have an excellent weight/mechanical properties ratio and a high degree of automation in their manufacture and recyclability. However, these materials present difficulties in their machining due to their nature. Their anisotropy, together with their low glass transition temperature, can produce important defects in their machining. A process able to machine these materials correctly by producing very small thermal defects is abrasive waterjet machining. However, the dispersion of the waterjet produces a reduction in kinetic energy, which decreases its cutting capacity. This results in an inherent defect called a kerf taper. Also, machining these materials with reduced thicknesses can increase this defect due to the formation of a damage zone at the beginning of cut due to the abrasive particles. This paper studies the influence of cutting parameters on the kerf taper generated during waterjet machining of a thin-walled thermoplastic composite material (carbon/polyurethane, C/TPU). This influence was studied by means of an ANOVA statistical analysis, and a mathematical model was obtained by means of a response surface methodology (RSM). Kerf taper defect was evaluated using a new image processing methodology, where the initial and final damage zone was separated from the kerf taper defect. Finally, a combination of a hydraulic pressure of 3400 bar with a feed rate of 100 mm/min and an abrasive mass flow of 170 g/min produces the minimum kerf taper angle.

## 1. Introduction

The use of composite materials in industry has generated a large number of publications and research. Within their wide classification, carbon fiber–reinforced (CFRP) or glass fiber–reinforced (GFRP) polymer matrix composites are the most interesting [1,2,3]. These materials have an excellent weight-to-mechanical-properties ratio and have been of great importance in recent years, especially in the aerospace and automotive sectors, although others, such as sport, wind energy, and construction, also make use of these composites [4].

However, the type of polymeric matrix used in these materials is thermoset. This generates a series of drawbacks in production and application of these materials, especially in terms of recyclability and processing times [5]. For this reason, there is an alternative to this kind of matrix. In recent years, carbon fibers have been combined with a thermoplastic polymer (CFRTP) to replace thermosets [6,7]. Due to their chemical composition, these polymers have a major advantage over thermosets, as they can be reshaped after curing. In addition, within the wide range of thermoplastic polymers, there are high-performance polymers. These are able to reach large service temperature ranges and achieve excellent impact resistance [8,9]. Also, compared to thermosets, CFRTPs have a high degree of automation in their manufacturing and recyclability. This makes these materials strategic for various industries, such as automotive, aeronautics, civil, or sports [10].

Thermoplastic polyurethane (TPU) stands out. It is an elastic elastomer that can be manufactured by various methods and subsequently machined. According to Biron et al. [11], these polymers have a high performance and a current consumption level. Due to this, they can provide a considerable number of combinations of physical properties that make them extremely flexible materials and adaptable to a multitude of uses [12]. 

However, due to their complex nature, they are highly difficult to machine in order to obtain their final geometry. The main disadvantage is due to their reduced glass transition temperature. In most conventional technologies, such as milling or drilling, the temperatures generated exceed the glass transition temperature of CFRTP, giving rise to defectology.

Masek et al. [13] carried out a study on milling CFRTPs with different cutting geometries. The thermoplastic matrix used was polyphenylene sulfide (PPS). When undergoing dry machining, i.e., in absence of cooling liquids, the temperatures reached softened the thermoplastic matrix. This boosted the excess removal of the thermoplastic matrix, leaving the reinforcement free in the form of a burr or fraying. This issue is also generated in the machining of composite materials with a thermoset matrix [14]. This, together with the abrasive and adhesive wear generated on the tool, makes conventional processes ineffective when machining CFRTPs.

However, there are non-conventional technologies that make it easier to machine materials that are difficult to machine with conventional technology. Abrasive waterjet machining (AWJM) is now of great interest for the machining of thermoset and thermoplastic composites due to its excellent flexibility and high material removal performance [15,16]. Its main advantage compared to other processes is the reduction of thermal defects during machining. In this sense, the temperatures reached are very low, and CFRTPs and CFRPs can be machined without matrix removal [17,18,19].

**Figure 1:**
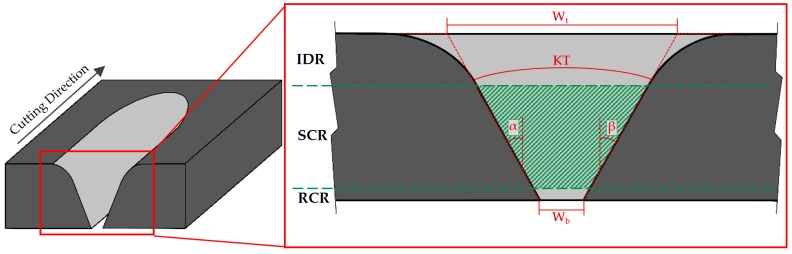
Detail of the cut section where it is appreciated: The initial damage region (IDR), the smooth cutting region (SMC), and the rough cutting region (RCR).

However, AWJM presents defectology due to the inherent nature of the process. The jet, when hitting the material, generates a reduction in kinetic energy. This produces a decrease in its cutting capacity, giving rise to a conical geometry known as a taper [20,21]. This is usually associated with two geometric factors (Figure 1)—Upper width (Wt) and lower width (Wb), giving rise to the angles α (°) and β (°). The sum of these angles gives the taper angle or the kerf taper (KT, Φ).

Nevertheless, the methodology for evaluation of conicity used in most articles can generate failures in their measurements. The formation of the zone known as the initial damage region (IDR) is due to the erosive action of abrasive particles, which produce a combination between this defect and the taper angle. In this way, the evaluation of upper width (Wt) can be increased due to this erosive action, giving rise to a dispersion in the evaluated taper, as observed in results shown in references [22,23,24].

Other researchers [20,24,25,26], however, have taken into account three regions with different defectology that may appear in the cut section (Figure 1). These are the upper region, called the initial damage region (IDR), where a greater erosive effect is produced due to the impact of the waterjet and abrasive particles on the surface of material; the central region, called the smooth cutting region (SCR), which presents a more homogeneous cut due to the stabilization of the waterjet on the cutting slot; and the lower region, called the rough cutting region (RCR), where the waterjet disperses again, losing a great deal of kinetic energy.

The taper defect is of great importance because it occurs in any material and can result in final geometries that do not have the required dimensions; it especially can result in reduced thicknesses.

Machining a composite material using AWJM creates a series of defects regardless of the matrix the composite is made of. The jet is conditioned by the change of materials that compose the composite. The matrix with which the composite is created will influence, to a greater or lesser extent, the generation of defects after machining. Authors such as Masek et al. [15] and Rao et al. [14] have similar conclusions after machining composites with different matrices. For that reason, due to the fact that CFRTP composition is close to CFRP, similarities can be established in their results.

Dhanawade et al. [27] carried out a study on abrasive waterjet cutting in a thermoset polymeric matrix composite material. The CFRP used was 26 mm thick. In this study, a response surface methodology was carried out in which an ANOVA statistical analysis was performed to determine the influence of the cutting parameters. Dhanawade established that the most influential parameter in the taper angle was hydraulic pressure. An increase in the kinetic energy of the waterjet is produced by increasing this parameter. This increases your cutting capacity and reduces the conicity of the cut. In this way, the kerf taper is directly influenced by the amount of impact of abrasive particles and kinetic energy given by the waterjet.

In addition, an increase in the feed rate of the machine decreases the overlap of abrasive particle impacts, reducing its cutting capacity and thus increasing the taper angle. Kinetic energy is also reduced with an increase in the distance between jet and the material—called the stand-off distance (SOD)—Because it generates a greater dispersion of the jet at exit.

The abrasive mass flow also has a high influence on the conicity generated during cutting. A small increase in this parameter decreases the conicity obtained due to the greater cutting capacity of the jet. However, an excessive increase in the amount of abrasive particles produces a collision between them, rounding their edges and reducing their cutting capacity, which generates a greater angle of conicity.

Similar results were obtained by El-Hofy [17]. In this study, the minimum conicity that can be obtained was indicated by applying high hydraulic pressure combined with a small stand-off distance. The conicity obtained is reduced by increasing the feed rate, contrary to the findings of Dhanawade et al. [27]. This is because, at high pressures, an increase in feed rate generates a smaller upper width (Wt), producing a more constant cut.

The distance between the focusing tube and the surface is a very important parameter for obtaining the proper conicity. This is mainly due to a loss of kinetic energy in the form of dispersion of the jet when leaving the focusing tube. Most studies indicate that a recommended distance is usually 2–3 mm [20,28,29].

Popan et al. [30] studied the influence of the variation of stand-off distances for a thickness of 6 mm. In this study, a reduction in this parameter of up to 0.5 mm reduced the upper cutting width (Wt), thus decreasing the taper. In addition, a reduction in stand-off distance produces a decrease in the radius of zone affected by erosion (IDR) due to the initial impacts of abrasive particles.

Also, the thickness of the material has a fundamental role in the conicity generated by machining. A reduction in material thickness enhances the influence of parameters considered less decisive in large thicknesses. Wong et al. [31] studied waterjet cutting in a thermoset composite material with a 3 mm thickness. In this study, hydraulic pressure and abrasive mass flow (AMF) take second place. In this way, the main parameter that affects the conicity of the cut is the combination of stand-off distance and feed rate. The combination of a high distance with a high feed produces maximum conicity.

In view of the above, more information is required on the influence of cutting parameters on conicity and on the reduction of this defect. In addition, as there is no literature focusing on waterjet machining with abrasive CFRTPs, it is necessary to determine the influence on matrix change [13]. Also, methodology established in most articles, gives rise to considerable errors in the assessment of this defect, by not separating it from the area affected by abrasive particles.

For these reasons, this article proposes the evaluation of taper angle using a new methodology based on image processing. In addition, influence of cutting parameters will be determined by means of an ANOVA statistical analysis, in order to discuss the results obtained in machining of thermoset composite materials. 

Finally, by means of a response surface methodology (RSM), a mathematical model will be obtained that predicts the conicity generated in abrasive waterjet cutting of low-thickness thermoplastic matrix composite materials.

## 2. Materials and Methods

In this article, carbon fiber (Twill 200 g/m^2^) was used as reinforcement, and thermoplastic resin (TPU, polyurethane) was used to manufacture the CFRTP composite. This composite was manufactured by a thermoforming process. Table 1 shows its main characteristics, as well as the fiber and matrix, respectively. 

A three-axis water-jet machine (TCI Cutting, BP-C 3020, Valencia, Spain) was used for the experimentation. The AWJM machine was equipped with an ultra-high capacity pump (KMT, Streamline PRO-2 60, Bd Nauheim, Germany). The water orifice of the machine had a diameter of 0.30 mm. The diameter and length of the focusing tube were 0.8 mm and 94.7 mm, respectively. All trials were carried out by a 120 mesh Indian garnet abrasive material.

In order to carry out the experimental design, a response surface methodology (RSM) was set up. This kind of methodology has already been employed by some authors in several experimental studies of the same order [27,31]. A face-centered composite design (FCD) with a total of 20 trials (8 factorial points–2^3^, 6 axial points–2 × 3, and 6 center points) was established and carried out using Minitab^®^ 18 software (18.1, Minitab, LLC, State College, PA, USA). 

A complementary experimental design was carried out. Because of this design, experimentally obtained data can be matched with data predicted by Minitab analytical software (18.1, Minitab, LLC, State College, PA, USA). This comparison makes it possible to obtain the error generated between these values and confirms the accuracy of the response surface model.

Three main parameters, which include hydraulic pressure, feed rate, and abrasive mass flow, were employed to determine their influence of the kerf taper generated. These parameters were designated based on the limitations of the CNC machine used, as well as the levels most employed in reviewed literature [17,22,24,31,32]. Also, they were converted into three different levels (–1, 0, 1) that represent minimum, central, and maximum values, respectively (Table 2). In order to establish a stand-off distance in accordance with the results of other authors [20,28,29], 2.5 mm was the stand-off distance used in all the tests.

**Figure 2:**
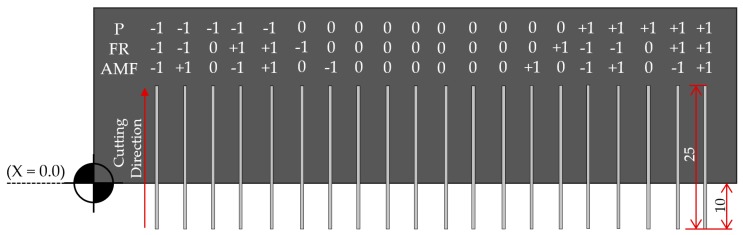
Experimental design scheme of CFRTP machining carried out.

Figure 2 shows the distribution of 20 test. They are machined in a 170 × 25 mm specimen with an 8 mm gap to optimize material consumption. Before machining, a horizontal cut was made at coordinate 0.0 in order to ensure the perpendicularity of each cut with the final machined part. A cutting length of 15 mm was fixed for each trial. Furthermore, each single cut starts 10 mm before the material side to achieve a constant flow of water and abrasive. Machining was carried out on three specimens (KT1, KT2, KT3) of the same CFRTP in order to obtain reproducibility of the results achieved.

On the basis of this methodology, an ANOVA analysis was developed in order to obtain the statistical influence of input parameters on output variables. Pressure, feed rate, and abrasive mass flow were changed in accordance with the fact that the experiment was conducted according to Box-Behnken design (three-level). In addition, RSM allows us to generate different contour diagrams or response surfaces from a second order polynomial Equation (1). There are several articles that have implemented this type of equation in order to develop the results obtained in the experiments carried out [31,33,34].

(1) $$Y=C_{0}+\overset{k}{\underset{i=1}{\sum }}C_{i}x_{i}+\overset{k}{\underset{i=1}{\sum }}C_{ii}x_{i}^{2}+\overset{k}{\underset{i<j}{\sum }}C_{ij}x_{i}x_{j}+\varepsilon$$

*Y* corresponds to the expected response, in this case the kerf taper generated (KT), *x*_*i*_ are the parameters used in the study (P, FR, AMF), *C*_0_, *C*_*i*_, *C*_*ii*_, *C*_*ij*_ are the regression coefficients, and *ε* is the random error of the model.

**Figure 3:**
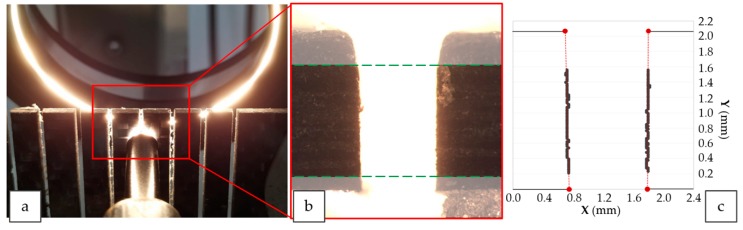
Macrographies obtaining procedure: (**a**) positioning in stereoscopical microscope; (**b**) image of a slot with the smooth cutting region (SCR) pointed out; (**c**) graph of the SCR points with intersection at 0.0 mm and 2.08 mm.

A stereoscopical microscope (Nikon, SMZ 800, Tokyo, Japan) was employed in order to obtain macrographies of each slot (Figure 3a). Image processing software made it possible to define the contour of the smooth cutting region (SCR) generated (Figure 3b). After machining, a first analysis was carried out to establish the range that delimits the three separate zones in section of cut. Later, the region obtained was split into 100 points. A trend line that adjust to those points was created. The intersection of this trend line with the value 0 mm (minimum thickness) and 2.08 mm (maximum thickness) was forced (Figure 3c).

These intersection points are used to obtain the top kerf width (Wt, mm) and the bottom kerf width (Wb, mm). Finally, the kerf taper defect (KT) is defined as shown in Equation (2), where *t* is thickness of CFRTP in millimeters.

(2) $$KT\;\left({}^{\circ}\right)=2*\mathrm{atan}\left(\frac{\frac{W_{t}-W_{b}}{2}}{t}\right)$$

## 3. Results

The kerf taper values obtained in the three specimens (KT1, KT2, KT3), as well as the average kerf taper and its standard deviation (average KT), are shown in Table 3. 

All the results of the mean values given in Table 3 are positive. This means that, according to Equation (2), the upper width of the cut is always greater than the lower width. It follows that the geometric shape obtained in all machined slots has a “V” shape. This geometric effect is produced by the material thickness together with the transverse feed rate and energy amount (pressure and focusing tube diameter). These variables affect the shape distortion of the slot [35]. It should be pointed out that the kerf taper defect generated in waterjet machining is independent of the thickness of material. This defect will be greater or smaller according to this thickness, but it will always happen, even in materials of small thicknesses, such as the CFRTP used in this article.

Average kerf taper values between 2.15° and 7.79° were obtained. It is necessary to remember that there is currently no article that analyzes the taper defect in abrasive waterjet machining of thermoplastic composite materials. Some of reviewed literature [22,24,27] shows values close to the range obtained in this experiment, although some specific kerf taper values are different. It must be taken into account that the composites used in the other articles are made of a thermoset matrix. Most of them contain a higher glass transition temperature than the thermoplastic resin used in this experiment. In addition, fixed variables such as the focusing tube diameter and abrasive grain size change during the mixing process and could generate different results.

On the other hand, not all of the existing literature takes into account the three areas generated in cutting slot—The initial damage region (IDR), the smooth cutting region (SCR), and the rough cutting region (RCR). The emphasis in this article is on the independent treatment of such areas. Several authors [17,22,24,27] calculate the kerf taper defect without taking this indication into account. This could cause the kerf taper values to be altered by the rounding radius generated on the top surface of machined material. This radius occurs when the waterjet hits the surface to be machined. Abrasive particles, in a first instant of contact, meet a wall that they must pass through. Not all of them are able to do it, so they disperse along the upper surface of the material, producing a rounding at the input of cut. 

Furthermore, taking into account the RCR zone means that there may be an error when obtaining the values. In this area, the waterjet comes out of machined material, resulting in a new opening. In composite materials, carbon fibers and the matrix can become detached by creating loose yarns or cavities. Therefore, 10% of the cutting slot as was chosen as the RCR zone.

**Figure 4:**
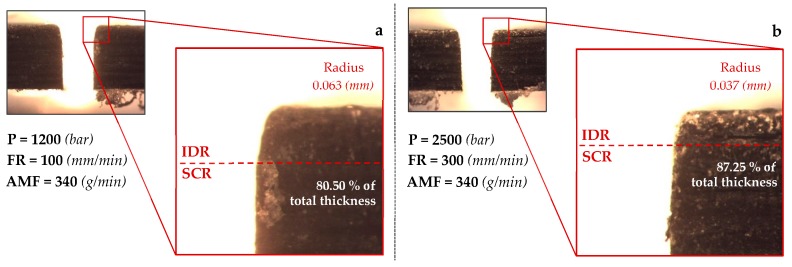
Cutting path macrograph for machining under conditions: (**a)**. P 1200 bar, FR 100 mm/min, AMF 340 g/min; (**b)**. P 2500 bar, FR 300 mm/min, AMF 340 g/min.

Two macrographies of the cutting section of two machined slots with different parameter combinations are shown in Figure 4. A rounded radius produced in the upper face of the cutting slot is shown in both figures. This defect is usually caused by the collision of the waterjet and abrasive particles with the top face of material. The radius obtained for Figure 4a is 0.063 mm, while the radius generated in Figure 4b is 0.037 mm.

This confirms the randomness that can be obtained in two slots machined on the same material. In addition, the point that separates IDR zone from SCR is found at different heights. Figure 4a shows the point at a height equivalent to 80.50% of total thickness (2.08 mm), and Figure 4b shows it at 87.25% of that thickness.

**Figure 5:**
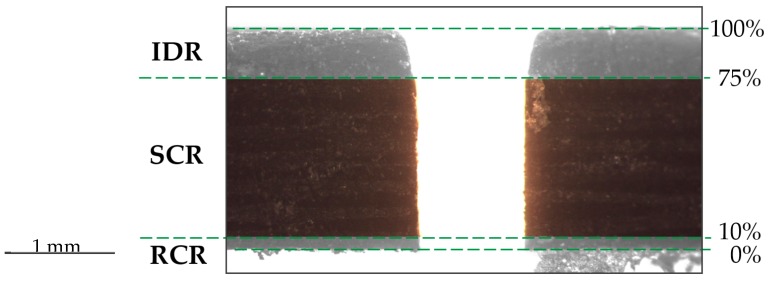
Detail of a real cut section where it can be seen the areas: Rough cutting region (RCR) 0–10%, smooth cutting region (SCR) 10–75%, initial damage region (IDR) 75–100%.

To sum up, taking into account the thickness of material used in this article, cutting section was split according to 0–10% of the thickness for the rough cutting region, 10–75% for the smooth cutting region and, 75–100% for the initial damage region. This article considers the kerf taper defect in the SCR (Figure 5). 

### 3.1. Statistical Analysis

An ANOVA analysis of the obtained kerf taper values is shown in the Table 4. As discussed in the methodology section, a second order quadratic model was employed. This model allows us to relate the input parameters used in experiment (pressure, feed, rate and abrasive mass flow) with the results obtained after machining. The ratio between both variables makes it possible to create greater or lesser accuracy between them. The p-value of this model is less than 0.05, which indicates that it is statistically significant. In addition, a high F-value will make variable more relevant in the analysis.

As can be seen in Table 4, pressure and feed rate are the most significant parameters in taper defect. This is deduced because p-value is 0, i.e., the significance is maximum. It is observed that the AMF parameter has a p-value of 0.15, which implies that this variable has less influence on the generation of the taper angle. In addition, it can be seen that the F-value for pressure variable triples to that obtained for feed rate. This allows us to deduce that, although both variables are significant, hydraulic pressure has greater weight in the generation of the kerf taper.

These statistical results are consistent with the articles related to the topic studied. Therefore, it can be concluded that, for generation of a taper defect, the order of parameters according to their influence is hydraulic pressure, feed rate, and abrasive mass flow.

Equation (3) shows mathematical model obtained for analyzed response surface methodology. This equation presents an R^2^ of 93.85%, which implies a value very close to 100%. In later sections, a verification of Equation (3) will be performed from input variables shown in Table 3.

(3) $$KT=1.00+(0.7P+124.8FR+153AMF+0.08FR^{2}-0.17AMF^{2}-0.04P*FR\;-0.13FR*AMF)*10^{-4}$$

### 3.2. Effect of Hydraulic Pressure on Kerf Taper

**Figure 6:**
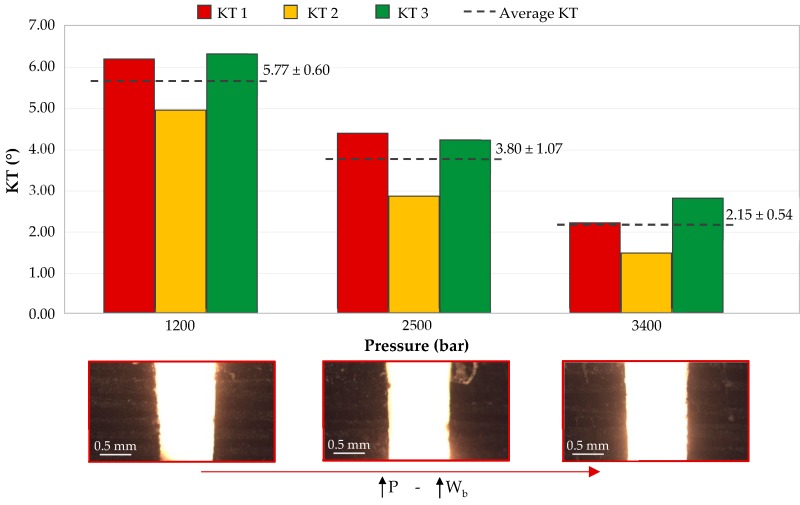
Variation of the kerf taper in three specimens as a function of pressure (FR = 300 mm/min; AMF = 225 g/min).

The effect of hydraulic pressure on variation of the kerf taper is shown in Figure 6. To discuss these data, feed rate and abrasive mass flow variables have been kept fixed and at their intermediate level (FR = 300 mm/min; AMF = 225 g/min). Three machined specimens have been classified with different colors. Thus, red color corresponds to test piece 1, yellow to test piece 2. and green to test piece 3. In addition, each figure shows the mean value of taper defect obtained for each test. This value was represented with a discontinuous line followed by mean value and resulting deviation. Pressure is one of the most influential variables in machining, therefore Figure 6 contains images that facilitate visual compression.

At first sight, the taper decreases as pressure increases. By taking a look at the mean values obtained and pressures involved, it can be seen how the resulting data adjust to a large extent to a linear regression. Higher pressure means an increase in kinetic energy generated by the waterjet. This increase in kinetic energy leads to a higher material removal. Consequently, the walls of the slot are subjected to a greater force in removing the material that causes a more vertical final state. 

**Figure 7:**
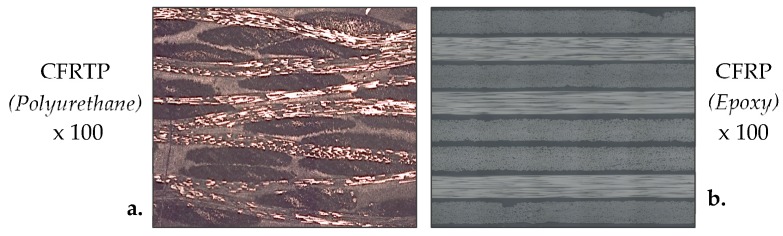
Macrographs of a composite cross-section with (**a**). thermoplastic matrix; (**b**). thermoset matrix.

By looking at the graph with values of each specimen independently, it can be seen how specimen 2 in all cases has a taper value smaller than 1 and 3. This may have been affected by the nature of the composite. The material is composed by long carbon fibers and thermoplastic matrix sheets. The arrangement of these elements along the composite is crucial to carry out the machining. In this way, a fiber yarn displaced at the time of manufacturing or an irregular consolidation of matrix along the surface could alter its homogeneity (Figure 7). Nevertheless, the decreasing tendency of the taper defect as hydraulic pressure increases is reflected in three specimens studied. In this case, a value of 3400 bar generates the lowest taper, being 2.15°. It should be noted that the thickness of composite employed is considered thin, which could make it easier for kinetic energy created by pressure to generate a homogeneous wall in slot.

A study carried out by Dhanawade et al. [27] shows a similar trend to that generated in this research. The material used in its study is a composite formed by carbon fibers and thermostable epoxy resin. They agree that an increase in pressure generates a decrease in taper defect. In addition, Dhanawade et al. use a hydraulic pressure that reaches 4000 bar, obtaining a taper angle that oscillates from 2.2° to 3.8°. It can be deduced that these results agree with those obtained in this article. Therefore, if the pressure parameter is analyzed in isolation, it seems that the kind of resin used in composite material does not greatly influence the kerf taper generated. 

Ruiz-Garcia et al. [20] carried out a study on the analysis of the kerf taper defect in the abrasive waterjet machining of CFRP/UNS A97075 stack. In that paper, the kerf taper results obtained were found in a range of 1°. Ruiz-Garcia et al. used lower feed rates than those used in this article. This resulted in a greater homogeneity of taper defects as well as a smaller variation of them. In addition, the matrix used by Ruiz-Garcia et al. was epoxy resin, which has a higher vitreous transition temperature than the thermoplastic resin used in this experiment (TPU—polyurethane, 145 °C). The use of a thermoset resin could allow the composite to achieve greater mechanical properties than the CFRTP employed in this experiment.

### 3.3. Effect of Feed Rate on Kerf Taper

**Figure 8:**
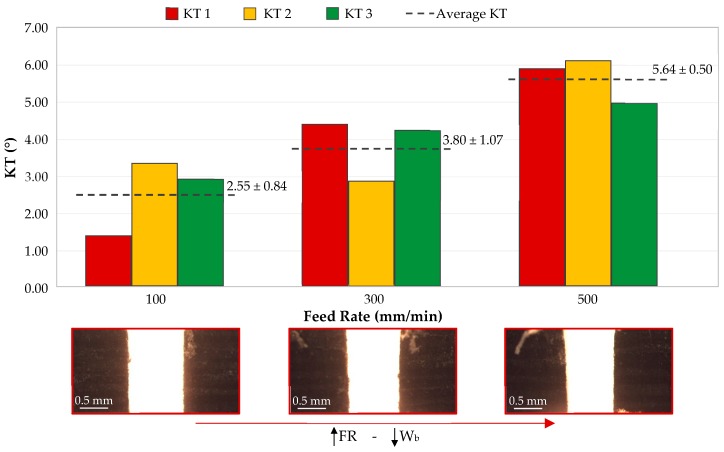
Variation of kerf taper in three specimens as a function of feed rate (P = 2500 bar; AMF = 225 g/min).

Figure 8 shows the effect of feed rate on the kerf taper defect. The distribution of elements in graph is similar to the one shown in Figure 6. As for previous section, this figure contains 3 images that make it easier to understand. It can be seen how, unlike for pressure, average values of kerf taper rise as feed rate increases. 

A slower cut is made when a slot is made with a minimum speed of 100 mm/min, causing a more homogeneous fracture along the surface of material. In this case, waterjet is able to pass through material and remove each layer of carbon fiber and matrix in an orderly way. This effect, together with abrasive particles, reduces the influence of cohesion nature of a composite material on the generation of kerf taper. Therefore, it follows that applying a low feed rate, kerf taper achieved will be smaller, or in other words, upper and lower width of slot are values closer to each other.

On the other hand, when a high feed rate is applied, a loss of kinetic energy is generated in cutting channel. These could produce a decrease in material removal and a decrease in abrasive particles affecting the surface of material. On this occasion, the waterjet is more susceptible to the nature of the material. Different layers of carbon fiber and matrix take advantage of the reduced energy of the jet to make it difficult to pass through the composite. This can lead to the slot walls not being perpendicular, resulting in the upper width of slot being greater than the lower. 

Thus, the lowest taper value will be produced for a feed rate of 100 mm/min, being 2.55°, and the highest value will be produced with a feed rate 500 mm/min, resulting in 5.64°. The lower thickness of the material employed, combined with a low feed rate, are parameters that help to create a homogeneous cut by AWJM.

Everything discussed above is consistent with Wong et al. [31]. In this paper, the authors analyze the taper defects in a carbon fiber thermostable matrix (epoxy). The FR used oscillated in 1000–2500 mm/min, which is a little bit higher than that carried out in this article. However, the authors conclude that a greater feed rate implies a lower amount of abrasive particles affecting the material. The decrease in the amount of these particles caused a dirtier and more random cut.

As the cutting speed increases, the upper and lower widths of the slot decrease. However, the width of the bottom surface has a greater decreasing tendency than the top surface. This is consistent with articles that study the taper defect in other kinds of materials [36,37].

### 3.4. Effect of Abrasive Flow Rate on Kerf Taper 

**Figure 9:**
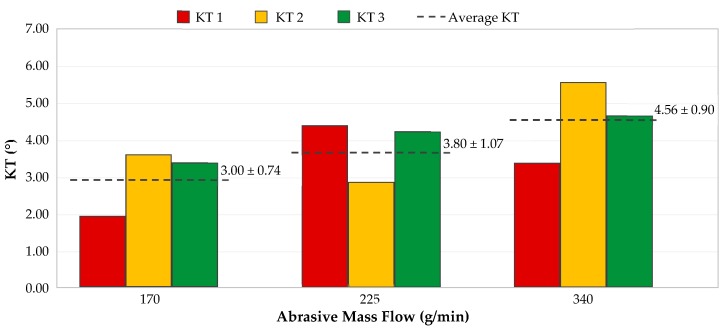
Variation of kerf taper in three specimens as a function of abrasive mass flow rate (P = 2500 bar; FR = 300 mm/min).

The influence of the abrasive mass flow (AMF) on generation of taper defect is shown in Figure 9. In Section 3.1, it was concluded that this parameter is the least influential of the three used in this article. However, this fact does not imply that the amount of abrasive employed does not affect the results. In fact, by looking at the trend of the mean values plotted in Figure 9, it can be seen how it ascends as the amount of abrasive increases. The upward trend caused by AMF is less than that caused by pressure and feed rate. Therefore, this parameter is the least influential of those used. 

It should be noted that an excessive increase in AMF can lead to a loss of kinetic energy. In this case, abrasive particles are more likely to collide with each other, causing a more disturbed cut, which translates into greater erosion at the input of slot, a greater difference between the upper and lower widths of the slot, and greater fraying at the outlet surface of the material [38].

In this case, a smallest kerf taper will be produced with a small amount of abrasive, 170 g/min, resulting in an angle of 3.00°. For the highest amount of abrasive, an angle of 4.56° is obtained.

Ruiz-Garcia et al. [20] achieved an upward trend similar to that obtained in this experiment. The use of a higher abrasive flow means a greater difference between the upper width of the slot and the lower width. Due to that, it can be noted that the abrasive flow rate used in AWJM is not linked to the kind of resin employed in the manufacture of composite.

### 3.5. Response Surface 

**Figure 10:**
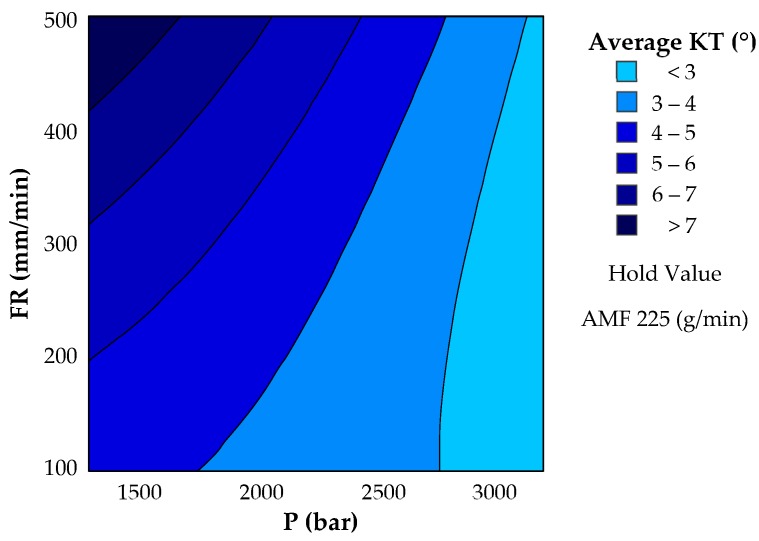
Contour plot of kerf taper defect as a function of most influential variables: Pressure and feed rate.

A response surface allows two input parameters to interact with an output variable, keeping all other parameters constant. The ANOVA analysis carried out in Section 3.1 gave hydraulic pressure and feed rate the greatest significance on the machining process. Therefore, FR and P have been represented together with the kerf taper in Figure 10, keeping AMF = 225 g/min fixed.

**Figure 11:**
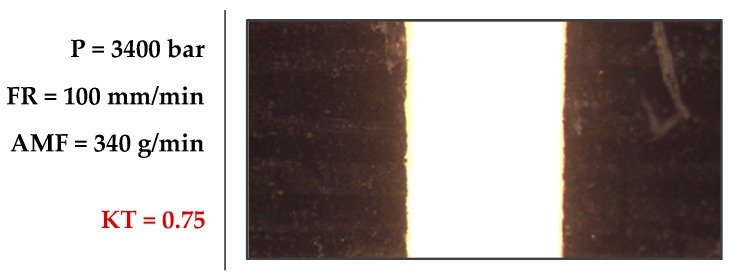
Combination that minimizes kerf taper defect (P = 3400 bar; FR = 100 mm/min; AMF = 340 g/min).

As can be seen in this figure, both parameters seem very significant. A value close to 3400 bar combined with various feed rate combinations offer the lowest kerf taper values (Figure 11). As a function of P-value, both P and FR were significant, while according to F-value, pressure tripled the influence of feed rate. This shows that a single pressure results in a range of kerf taper values (<3°) for the whole range of feed rates used in this experiment.

On the other hand, a combination of 1200 bar of pressure and 500 mm/min of feed rate seems to be the most unfavorable in the study of kerf taper, and it seems to be the element that greatly increases the upward trend. As speed increases, cutting capacity decreases due to the loss of kinetic energy in the waterjet. To this effect, it would be necessary to add the reduction of the number of abrasive particles that affect the material.

In addition, the polyurethane matrix applied in this article has a melting temperature of 145 °C. Abrasive waterjet cutting is a technology that generates a lower temperature than other conventional machining technologies, such as milling or drilling [14]. This process makes it easier to machine temperature-sensitive materials. High temperatures could lead to thermoplastic matrix softening, generating defects in the final quality of the slot. The kerf taper values achieved are similar to those studied by Wong et al. [31]. This could mean that the effect of temperature on the thermoplastic matrix is not enough to alter the kerf taper.

According to our mathematical model, a combination of P = 3400 bar, FR = 172.73 mm/min, and AMF = 170 g/min should result in the lowest kerf taper value of 1.79°. The degree of desirability of the result achieved is also obtained. Individual and compound desirability evaluates how well a combination of parameters satisfies the objectives defined for output variables.

Individual desirability (d) evaluates how adjustments optimize a single response. The compound (D), on the other hand, looks at how adjustments optimize a set of responses in general. This variable has a range of 0 to 1, 1 being the ideal case, and 0 meaning that some of answers obtained are outside acceptable limits. In this case, a maximum desirability was generated, with a value of 1, which implies that the combination of cutting parameters selected offers a desirable result.

### 3.6. Mathematical Model Validation

Table 5 shows the taper values obtained from the complementary experimental design together with the same values predicted by Equation (3). 

**Figure 12:**
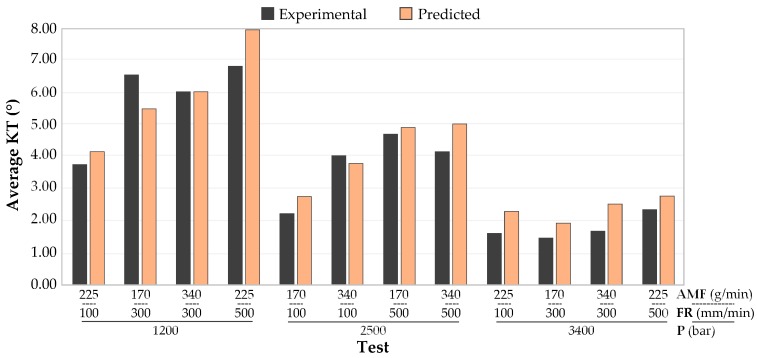
Kerf taper values (experimental and predicted).

In order to evaluate the mathematical model carried out in this experiment, a comparison between combinations of parameters not used in original DOE and predicted values was carried out (Figure 12).

It should be noted that, in most cases, experimental values are below those predicted at a similar distance. When the errors tabulated in Table 5 are observed, it can be seen that, for pressures of 1200 and 2500 bar, errors oscillate in a range of 0–20%, while highest pressure of 3400 bar generates high errors with values of up to 49.84%. 

The average error obtained is 20.49%. Experimental values follow the trend of the predicted values but maintain an almost constant difference between them. This difference may be due to the anisotropy and nature of material, as seen in Figure 7. This model could be used to predict the trend that will follow the kerf taper as a function of parameters used, although the error obtained must be taken into account.

## 4. Conclusions

An experimental study on the influence of cutting parameters on the generation of a geometric defect, called a kerf taper, focused on the machining of carbon fiber composite materials with thermoplastic matrix, was developed. A face-centered composite experimental design (FCD) based on a response surface methodology (RSM) was development. This type of experimental design has given rise to a second order polynomial equation that relates input parameters to output variable (kerf taper).

The literature review allowed for the selection of pressure, feed rate, and abrasive mass flow as the most influential input parameters in the machining performed while keeping other parameters, such as stand-off distance or size of abrasive particles, fixed.

A second experimental design was used to verify the second order polynomial equation generated by the taper obtained, which contrasts combinations of parameters not used in the original DOE with values predicted by model. An error of 20.49% was obtained, which can be considered small if the anisotropy and nature of a composite material are taken into account.

Three zones along thickness of material have been identified—The rough cutting region at 0–10%, the smooth cutting region at 10–75%, and the initial damage region at 75–100%. In addition, only the Smooth Cutting Region was taken into account for the development of this experiment.

The kerf taper defect was studied in three specimens of the same thermoplastic composite material in order to obtain an average value and its respective deviation. Marginal graphs show how hydraulic pressure causes a decrease in taper generated, and feed rate and abrasive mas flow produce an increase in the same.

ANOVA analysis has indicated that hydraulic pressure and feed rate are the most influential parameters in abrasive waterjet machining. The slot walls become more vertical at high pressures and low feed rates. This is due to a higher concentration of energy impacting the composite to be machined, which translates into higher material removal. Also, the mathematical model obtained for analyzed response surface methodology has presented an R^2^ of 93.85%.

The effect of temperature does not seem to influence the quality of the results obtained by AWJM. After a concise literature review, it seems that the results obtained in taper defect agree with those obtained by other scientific authors.

Finally, a combination of cutting parameters that minimizes kerf taper defect was found, resulting in a pressure of 3400 bar, a feed rate of 100 mm/min, and an abrasive mass flow of 340 g/min, producing an upper-lower width ratio close to 1, i.e., 0.75°. This small kerf taper defect means that for specific applications of AWJM could be considered as a high precision process.

## Figures and Tables

**Table 1:** Fiber, matrix, and carbon fiber–reinforced thermoplastic (CFRTP) characteristics.

**Reinforcement**	Fiber type	Twill 200 g/m^2^
	Fiber thickness	0.25 mm
**Matrix**	Resin type	TPU (Polyurethane)
	Melting Temperature	145 °C
**CFRTP**	Ply Orientation	[0°/90°]s
	Number of plies	7
	Composite thickness (t)	2.08 mm
	Fiber/Matrix (%)	74.2/25.8

**Table 2:** Cutting parameters set.

Parameter	Symbol	Units	Level −1	Level 0	Level 1
Hydraulic Pressure	P	bar	1200	2500	3400
Feed Rate	FR	mm/min	100	300	500
Abrasive Mass Flow	AMF	g/min	170	225	340
Stand-off distance	SOD	mm	Fixed at 2.5		

**Table 3:** Kerf taper values obtained in all tests.

Test	Pressure [bar]	Feed Rate [mm/min]	Abrasive [g/min]	KT 1 [°]	KT 2 [°]	KT 3 [°]	Average KT [° ± (°)]
1	1200	100	170	4.71	3.23	3.92	3.95 ± 0.60
2	1200	100	340	4.09	5.25	4.67	4.67 ± 0.47
3	1200	300	225	6.14	4.92	6.25	5.77 ± 0.60
4	1200	500	170	6.51	7.18	9.58	7.76 ± 1.32
5	1200	500	340	7.53	7.80	7.73	7.69 ± 0.11
6	2500	100	225	1.39	3.34	2.91	2.55 ± 0.84
7	2500	300	170	1.96	3.62	3.41	3.00 ± 0.74
8	2500	300	225	2.43	3.43	3.13	3.00 ± 0.42
9	2500	300	225	5.12	2.16	6.39	4.55 ± 1.77
10	2500	300	225	4.22	2.03	5.98	4.07 ± 1.62
11	2500	300	225	4.77	2.26	3.25	3.43 ± 1.03
12	2500	300	225	5.12	2.85	3.16	3.71 ± 1.00
13	2500	300	225	4.54	4.40	3.26	4.07 ± 0.57
14	2500	300	340	3.39	5.59	4.70	4.56 ± 0.90
15	2500	500	225	5.88	6.10	4.94	5.64 ± 0.50
16	3400	100	170	1.61	2.11	3.08	2.27 ± 0.61
17	3400	100	340	0.75	2.91	4.84	2.83 ± 1.67
18	3400	300	225	2.16	1.48	2.81	2.15 ± 0.54
19	3400	500	170	1.52	2.69	3.31	2.51 ± 0.74
20	3400	500	340	1.68	2.16	3.68	2.51 ± 0.85

**Table 4:** ANOVA analysis of the kerf taper values obtained.

Source	DF	Adj SS	Adj MS	F-Value	*p*-Value
Model	9	48.2278	5.3586	16.95	0.00
Pressure (bar)	1	30.6617	30.6617	97.00	0.00
Feed Rate (mm/min)	1	9.8658	9.8658	31.21	0.00
Abrasive (g/min)	1	0.7668	0.7668	2.43	0.15
Error	10	3.1610	0.3161		
Lack-of-Fit	5	1.6498	0.3300	1.09	0.46
Pure Error	5	1.5112	0.3022		
Total	19	51.3888			

**Table 5:** Complementary DOE for validation of the mathematical model.

Test	Pressure [bar]	Feed Rate [mm/min]	Abrasive [g/min]	KT [°]	Predicted KT [°]	Error [%]
1	1200	100	225	3.73	4.13	10.81
2	1200	300	170	6.52	5.46	16.21
3	1200	300	340	5.99	5.98	0.12
4	1200	500	225	6.77	7.95	17.47
5	2500	100	170	2.23	2.75	23.19
6	2500	100	340	4.01	3.76	6.39
7	2500	500	170	4.68	4.89	4.37
8	2500	500	340	4.17	5.00	19.85
9	3400	100	225	1.60	2.28	42.33
10	3400	300	170	1.40	1.91	36.27
11	3400	300	340	1.67	2.50	49.84
12	3400	500	225	2.32	2.76	19.07
